# Invasive Ductal Breast Cancer with Osteoclast-Like Giant Cells: A Case Report Based on the Gene Expression Profile for Changes in Management

**DOI:** 10.3390/jpm11020156

**Published:** 2021-02-23

**Authors:** Azzurra Irelli, Maria Maddalena Sirufo, Gina Rosaria Quaglione, Francesca De Pietro, Enrica Maria Bassino, Carlo D’Ugo, Lia Ginaldi, Massimo De Martinis

**Affiliations:** 1 Medical Oncology Unit, Department of Oncology, AUSL 04 Teramo, 64100 Teramo, Italy; azzurra.irelli@hotmail.it; 2 Department of Life, Health and Environmental Sciences, University of L’Aquila, 67100 L’Aquila, Italy; maddalena.sirufo@gmail.com (M.M.S.); fra722@hotmail.it (F.D.P.); enricamaria.bassino@gmail.com (E.M.B.); lia.ginaldi@cc.univaq.it (L.G.); 3 Allergy and Clinical Immunology Unit, Center for the diagnosis and treatment of Osteoporosis, AUSL 04 Teramo, 64100 Teramo, Italy; 4 Pathological Anatomy Unit, Mazzini Hospital, AUSL04 Teramo, 64100 Teramo, Italy; gina.quaglione@aslteramo.it; 5 Radiotherapy Unit, Department of Oncology, AUSL 04, 64100 Teramo, Italy; carlo.dugo@aslteramo.it

**Keywords:** rare breast cancer, osteoclast-like giant cells, gene profiling, Oncotype Dx, adjuvant treatment

## Abstract

We report the case of a 49-year-old woman diagnosed with a rare histotype of early breast cancer (BC), invasive ductal carcinoma with osteoclast-like giant cells (OGCs), from the perspective of gene profile analysis tests. The patient underwent a quadrantectomy of the right breast with removal of 2 cm neoplastic nodule and three ipsilateral sentinel lymph nodes. The Oncotype Dx gave a recurrence score (RS) of 23, and taking into account the patient’s age, an RS of 23 corresponds to a chemotherapy benefit of 6.5%. After a multidisciplinary collegial discussion, and in consideration of the patient’s age, the absence of comorbidity, the premenopausal state, the rare histotype and the Oncotype Dx report, the patient was offered adjuvant chemotherapy treatment followed by hormone therapy. This case may be an example of the utility of integrating gene expression profiling tests into clinical practice in the adjuvant treatment decision of a rare histotype BC. The Oncotype Dx test required to supplement the histological examination made us opt for the proposal of a combined treatment of adjuvant chemotherapy followed by adjuvant hormone therapy. It demonstrates the importance of considering molecular tests and, in particular, the Oncotype Dx, in estimating the risk of disease recovery at 10 years in order to identify patients who benefit from hormone therapy alone versus those who benefit from the addition of chemotherapy, all with a view toward patient-centered oncology. Here, we discuss the possible validity and limitations of the Oncotype Dx in a rare luminal A-like histotype with high infiltrate of stromal/inflammatory cells.

## 1. Introduction

Breast cancer (BC) represents the most frequently diagnosed cancer among women regardless of age. Male BC is rare and affects about 1% of cases [1,2,3,4].

In Italy, about 55,000 new cases of BC have been reported among women in 2020, and there are 834,200 women who survive BC after a diagnosis. To date, BC represents the leading cause of death from cancer among women, with over 12,300 deaths in all age groups, although mortality is declining in all age groups, especially in women under the age of 50, probably due to the spread of screening programs and to therapeutic progress. The 5-year survival of women with breast cancer BC patients is 87%, while survival 10 years after diagnosis is 89% [5].

Overall, BC accounts for 30% of female cancers among women and is the leading cause of cancer death among women between the ages of 20 and 59. Cancer screening programs and advances have resulted in a steep drop in BC mortality, so much so that, as of 2017, the death rate has dropped from its peak in 1989 by 40% [6]. BC diagnosis is based on clinical examination, radiological imaging and histological type.

The two most frequent subtypes of invasive BC are carcinoma not otherwise specified (70–75% of cases) and lobular carcinoma (12–15%). The other 18 subtypes exhibit specific morphological traits and are rare (0.5–5%) [7].

Invasive carcinomas have been classified by histological subtype as “favorable” (mucinous, tubular, cribriform, tubulo-lobular and lobular) and “unfavorable” (ductal, mixed ductal and lobular and micropapillary carcinoma). The histological subtypes with the highest percentage of high recurrence score (RS) were invasive micropapillary, pleomorphic lobular and ductal carcinoma [8].

The indication for systemic adjuvant therapy is decided on the basis of the biological characteristics of the tumor (the histological type, presence or absence of ductal carcinoma in situ, grade, Ki67, presence of peritumor vascular invasion, receptors for estrogen (ER), receptors for progesterone (PR), human epidermal growth factor receptor 2 (HER2) status, the number of regional lymph nodes involved, dimensions) and the patient’s clinical characteristics (age, performance status, comorbidity), with the help of scales such as Activities of Daily Living (ADL), Instrumental Activities of Daily Living (IADL), Cumulative Illness Rating Scale (CIRS) and, considering the toxicities of the proposed therapy, the patient’s life expectancy, as well as her preferences [9,10,11,12,13]. 

Multigenic prognostic tests help to identify hormone receptors (HR)-positive, luminal-like and HER2-negative early BC patients who could benefit from chemotherapy, providing an estimate of the risk of recurrence after 10 years [1]. 

Among different kind of tests, the Oncotype Dx, a molecular test that uses quantitative reverse transcription polymerase chain reaction (qRT-PCR) technology, has both a prognostic value for 10-year risk of recurrence and a predictive value in terms of survival advantage from adjuvant chemotherapy. This test was built as a mathematical model based on the analysis of 21 genes (16 genes that inform about the proliferative state of the tumor and 5 control genes) and allows for division of the operated items for early breast cancer into risk categories. By assessing the differential expression of these genes, it is possible to associate gives each tumor with a score from 0 to 100. The score is called the recurrence score (RS) and predicts the risk of distant relapse within 10 years in patients with luminal/HER2-negative tumor. A higher RS is associated with a greater risk of distant cancer relapse in the 10 years following the diagnosis of early BC [9,14,15].

A higher RS was observed in invasive ductal carcinoma with micropapillary features, followed by invasive ductal carcinoma not otherwise specified, invasive mucinous carcinoma, invasive lobular carcinoma, mixed ductal and lobular carcinoma, tubular carcinoma, mixed and mucinous ductal carcinoma and pleomorphic lobular carcinoma. For special histological types of BC, it is unclear whether RS is as significant as in nonspecial type carcinomas [16].

In particular, the prevalence of high RS has been observed to be lower in BC patients with lobular than in those with non-lobular histotype [17,18].

The combination of genomic and clinical information provides the clinician with a more accurate estimate of the BC patient’s prognosis than considering genomic or clinical information, alone [19].

BC with osteoclast-like giant cells (OGCs) was first described by Leroux in 1931; then by Duboucher in 1933 and Factor in 1977 [20] and subsequently by Agnatis in 1979 [21], Holland in 1984 [22] and Pettinato in 1989 [23].

The origin and nature of multinucleated OGCs in extra-skeletal tumors are not defined. OGCs are a specific type of macrophage different from osteoclasts. Bone resorption by OGCs isolated from breast tissue and the breast indicates that this transplanted cell into new tissue performs the bone resorption function of the osteoclast [24]. 

BC with OGCs is a rare histotype found in 0.5–1.2% of BC cases, with an unknown OGC mechanism of formation.

This histotype is characterized by the presence of OGCs, or giant cells similar to multinucleated osteoclasts, in association with ductal, lobular, papillary, cribriform, tubular, mucinous, scaly or other BC [25,26,27]. 

Among the histological types of breast cancer with OGCs reported, invasive ductal carcinoma is the most frequent histotype reported in association with OGCs [28], particularly, the luminal-like A subtype [29].

OGCs have similar characteristics to bone osteoclasts but have lost antigen presentation capabilities, such as an anticancer defense. The appearance of OGCs could result from a protumor differentiation of macrophages that respond to hypervascular microenvironments induced by BC. OGCs correspond to cells that strongly express the pan-macrophage marker CD68 and variably express CD163, a marker of the M2-macrophage with protumor function.

The high content of tumor-associated macrophages (TAMs) in the BC microenvironment is associated with a worse prognosis [30,31,32].

Genomic tests investigate genes associated with the proliferation and estrogen receptors of cancer cells for risk stratification but do not consider the tumor microenvironment, hence the perplexity of using these tests for tumors with special histology, particularly if they are rich in macrophages [33].

## 2. Case Report

We reported the case of an early BC, invasive ductal carcinoma with OGCs, from the perspective of gene profile analysis tests.

At the end of February 2020, a 49-year-old nonsmoking female patient with no comorbidity and unfamiliar with oncological diseases, underwent a screening mammography x-ray that showed the presence of a nodule of about 2 cm against the external quadrant of the right breast, which was suspected for heteroplasia in the absence of further suspect nodules and/or lymph nodes. The patient was then subjected to an ultrasound examination, which confirmed the presence of a lump with malignant characteristics, and was therefore subjected to true-cut of the breast lump with histological examination positive for invasive ductal carcinoma of the breast.

In March 2020, the patient underwent a quadrantectomy of the right breast with removal of 2 cm neoplastic nodule and three ipsilateral sentinel lymph nodes. The microscopic examination was positive for moderately differentiated invasive ductal carcinoma containing osteoclast-like giant cells (OGCs). According to immunohistochemical analysis, the tumor had the following characteristics: ER: + 100%, PR: + 85%, E-cadherin: positive, Ki67: + 10% and HER2: negative, with a staging category corresponding to pT1c pN0, according to the TNM staging system. 

Immunohistochemically, OGCs are positive for the histiocytic marker CD68 and negative for E-cadherin, an epithelial marker, and for ER, PR and HER2. In addition, they are CD163 positive (Figure 1, Figure 2, Figure 3, Figure 4, Figure 5, Figure 6, Figure 7 and Figure 8).

On the other hand, cancer cells are negative for CD68 and CD163 for the HER2 but are positive for cadherin E, estrogen receptor (ER: 100%) and progesterone receptor (PR: 85%) (Table 1).

Clinical–instrumental staging tests (blood chemistry tests with tumor markers, abdomen ultrasound, chest CT without bone and bone scan) were negative for distant neoplastic disease.

For the negative HER2 hormone-responsive disease, various analysis tests of gene profiles are available and are useful in determining the risk of relapse of disease in early breast cancer, in order to assess the need for chemotherapy in addition to hormone therapy.

In this clinical case, the Oncotype Dx test provided was used in our institute, with a recurrence score of 23. Considering the age of the patient, ≤50 years, the use of chemotherapy was found to correspond to a benefit of approximately 6.5%.

After a multidisciplinary collegial discussion, and considering the age, the absence of comorbidity, the premenopausal state, the rare histotype and the Oncotype Dx report, the patient underwent adjuvant chemotherapy treatment, according to the Docetaxel-Cyclophosphamide q21 scheme, for 4 cycles, followed by hormone therapy with luteinizing hormone-releasing hormone (LHRH) analogue and exemestane.

The patient, aware of the benefits and risks related to the aforementioned therapeutic proposal, decided to accept it. After chemotherapy, the patient will also undergo radiotherapy treatment on the residual breast.

## 3. Discussion

In several other diseases, such as tuberculosis, sarcoidosis and granulomatous mastitis, we can found the presence of OGCs. However, breast cancer does not have histological features consistent with granulomatous disease. The origin and mechanism for developing osteoclast-like giant cells is unknown. However, one hypothesis suggests that cancer cells secrete the vaso-endothelial growth factor, which promotes angiogenesis and migration of macrophages into the tumor; this eventually induces monocytic stromal cells to merge with each other to become OGCs [34].

Immunohistochemical studies suggest that OGCs originate from mesenchymal cells, particularly macrophages, in response to cytokines produced by cancer cells [28].

The secretion of cytokines, such as VEGF and MMP12, indeed determines an inflammatory and hypervascular stroma and improves macrophage migration. Therefore, the appearance of OGCs could be not an antitumor immunological reaction but a differentiation of macrophages that respond to the hypervascular tumor microenvironment induced by breast cancer. OGCs have a phenotypic similarity to osteoclasts in the bone and lack antigen presentation capabilities [30].

It has also been shown that when OGCs are isolated from breast carcinomas BC and placed in cell cultures on bone slices, they perform an osteoclast function with consequent formation of bone resorption pits [20].

While osteoclasts are activated by osteoblasts, OGCs are activated directly by the presence of the parathyroid hormone. Furthermore, OGCs are not inhibited by calcitonin, demonstrating another key distinction between OGCs and osteoclasts [35].

The histopathological diagnosis of BC with OGCs passes through the immunohistochemical determination of markers such as E-cadherin, CD68 and CD163. E-cadherin stains tumor cells but not OGCs; CD68 stains OGCs but not tumor cells; CD163 is expressed inconsistently by OGCs, i.e., with moderate- to high intensity [31], or is not expressed [32,36]. In the clinical case we present here, CD163 stains both macrophages and OGCs. 

Due to the limited number of cases of this rare histological subtype in clinical practice, it is difficult to establish the prognosis in these patients [20].

The prognostic significance of the presence of OGCs in breast cancer remains controversial, as some authors have suggested a less favorable prognosis for invasive breast cancer with OGCs among BC [14], while others have reported a similar or better prognosis than infiltrative carcinomas without OGCs.

Given this discrepancy, the prognosis in these patients is much more likely associated with the BC histology than with the presence or absence of OGCs [26,28,35].

With the above doubts regarding the prognosis of this tumor, we decided to use the Oncotype Dx test for the patient under consideration.

The Oncotype Dx is used in early luminal-like and HER2-negative BC patients [37].

In the TAILORx study. Sparano et al. have enrolled 6711 early BC patients with hormonal-positive receptors, HER2 negative and without locoregional lymph node metastases. The Oncotype Dx allowed patients to be stratified into three groups based on the RS value: patients with RS ≤ 10 underwent exclusive hormone therapy; patients with RS > 25 underwent chemotherapy followed by hormone therapy and patients with RS = 11–25 were randomized to receive hormone therapy versus chemotherapy followed by hormone therapy. It was observed that, for patients with RS 16–25, the combination of RS and age <50 years identifies patients who benefit from the addition of chemotherapy to hormone therapy: for RS 16–20, approximately 1.6% benefit from chemotherapy; for RS 21–25, approximately 6.5% benefit from chemotherapy [38,39].

The Italian prospective study ROXANE assessed the impact of the Oncotype Dx in clinical practice in nine Italian cancer centers. This test was used when the recommendation of adjuvant treatment with chemotherapy was uncertain for 251 patients with early luminal-like/HER2 negative BC, T1-T3, N0-N1. The rate of change in the treatment decision was 30% (n = 75), mainly from chemotherapy plus hormone therapy to hormone therapy (76%, n = 57/75). The proportion of patients recommended to chemotherapy plus hormone therapy (n = 130) was significantly reduced from pre-RS to post-RS (from 52% to 36%, *p* < 0.0001). Among the 121 BC patients, candidates for exclusive hormone therapy without the Oncotype Dx, 18 (15%) patients obtained an RS that referred to chemotherapy treatment followed by hormone therapy. The percentage of patients initially recommended for hormone therapy alone for whom the recommendation changed to chemotherapy plus hormone therapy was low (7%) [40].

We reported the case of a patient with rare and grade 2 luminal A-like BC. Given the rarity of the histotype, the case was subjected to a second anatomopathological review at another hospital, which confirmed the histopathological characteristics reported. Given that luminal A-like tumors are more likely low-grade and with low RS than luminal B-like tumors [41], by subjecting this case to Oncotype Dx, we expected to obtain a low RS. We obtained an intermediate RS of 23, which, combined with the patient’s age of 49, suggested a chemotherapy benefit of approximately 6.5%. The reliability of this result is questioned by the studies of Acs G. et al. [42,43], who recognized the inflammatory cells of the tumor microenvironment as factors influencing the RS by increasing it. For example, Mammostrat, an immunohistochemistry-based assay that analyzes only tumor cells, could represent a valid alternative to the Oncotype Dx that analyzes RNA extracted from both tumor cells and stromal/inflammatory cells in cases of BC with inflammatory infiltrate. In fact, tumors with intermediate/high risk in the Oncotype Dx but not with Mammostrat showed a tumor microenvironment rich in inflammatory cells. We can deduce that, in the case of inflammatory tumors, the Oncotype Dx could have less informative value, but further studies are needed to validate this hypothesis. However, it should be remembered that Mammostrat is not currently available on the market in Italy [15].

## 4. Conclusions

We presented a clinical case of early breast cancer with a rare histotype for which we used one of the gene expression profiling tests available, i.e., Oncotype Dx, in order to identify the best therapeutic procedure for the patient. Based exclusively on histopathological parameters, except histology, we would have offered the patient exclusive hormonal treatment. The Oncotype Dx together with the age of the patient and her premenopausal state, as well as the rare histology, made us opt for adjuvant chemotherapy followed by adjuvant hormone therapy. It follows the importance of considering molecular tests and, in particular, prospectively validated genomic tests such as Oncotype Dx, with the limits related to the literature data available on special histologies, in estimating the risk of disease recovery at 10 years, in order to identify the best treatment with a view to personalized, patient-centered oncology.

## Figures and Tables

**Figure 1:**
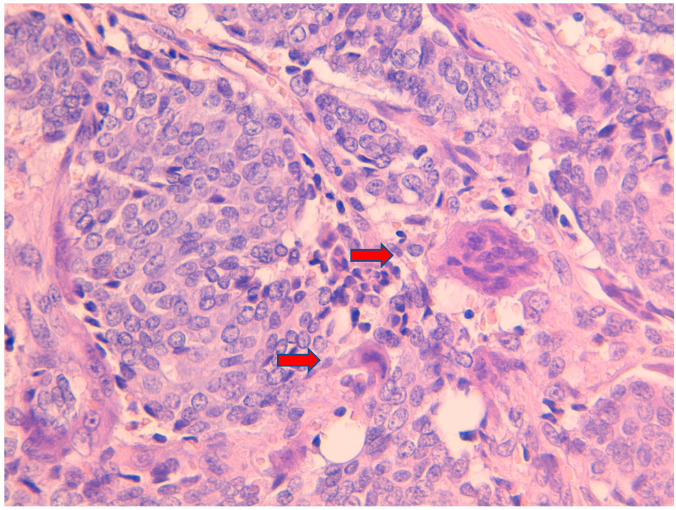
Hematoxylin and eosin stain 40× showing invasive ductal carcinoma with at least 2 osteoclast-like giant cells (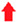).

**Figure 2:**
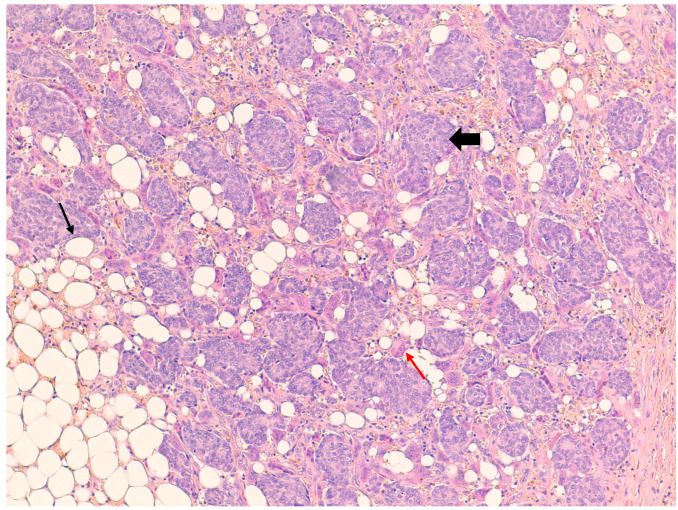
Hematoxylin and eosin stain 20× showing invasive ductal carcinoma with at least 30 osteoclast-like giant cells (↑), which are multinucleated cells and vary in shape and size with eosinophilic cytoplasm; grouped neoplastic cells (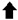) are present immersed in the stroma containing adipocytes (↑).

**Figure 3:**
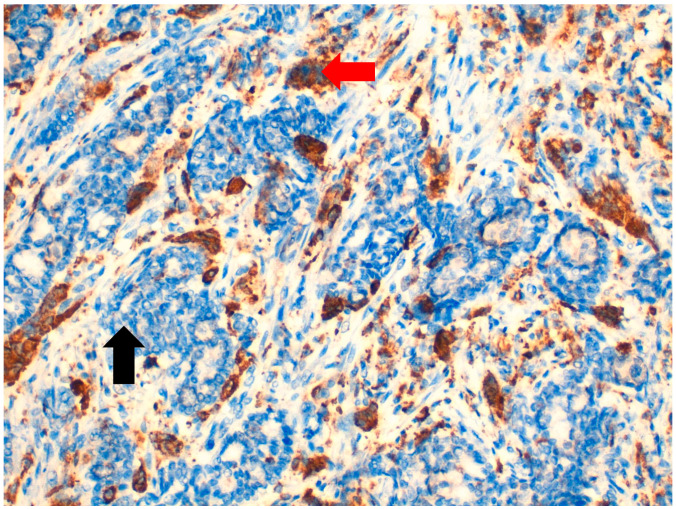
At 40× magnification. Immunohistochemical-positive reaction of macrophages included osteoclast-like giant cells (OGCs) for CD68 (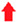), unlike neoplastic cells, which are CD68-negative (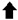).

**Figure 4:**
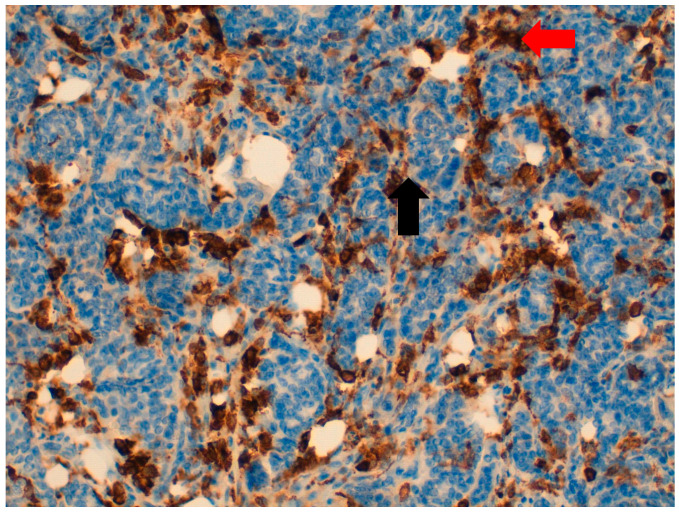
At 40× magnification. Immunohistochemical-positive reaction of OGCs and macrophages for CD163 (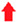), unlike neoplastic cells, which are CD163-negative (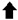).

**Figure 5:**
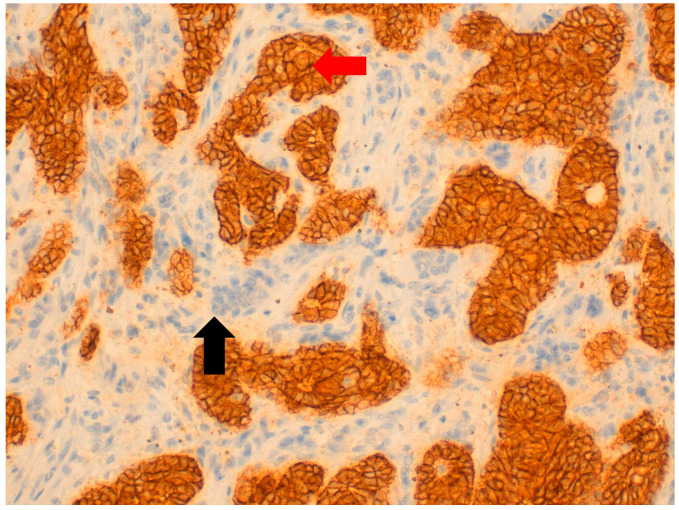
At 40× magnification. Immunohistochemical-positive reaction of neoplastic cells for E-cadherin (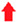), unlike OGCs, which are E-cadherin-negative (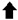).

**Figure 6:**
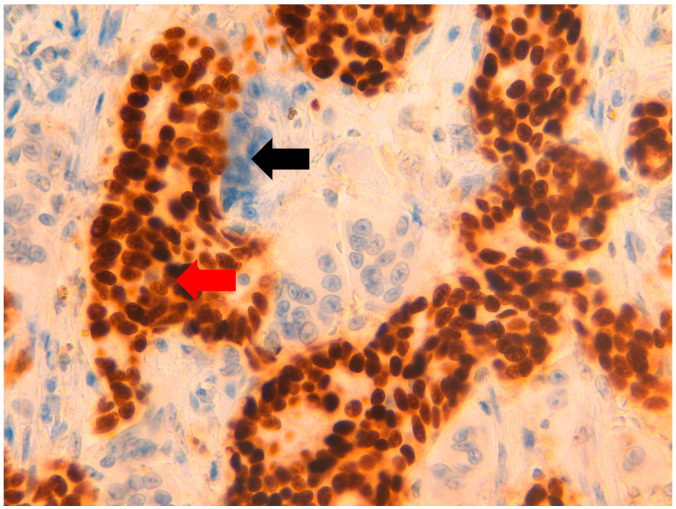
At 40× magnification. Immunohistochemical-positive reaction of neoplastic cells for estrogen receptor (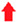), unlike OGCs, which are estrogen receptor-negative (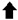).

**Figure 7:**
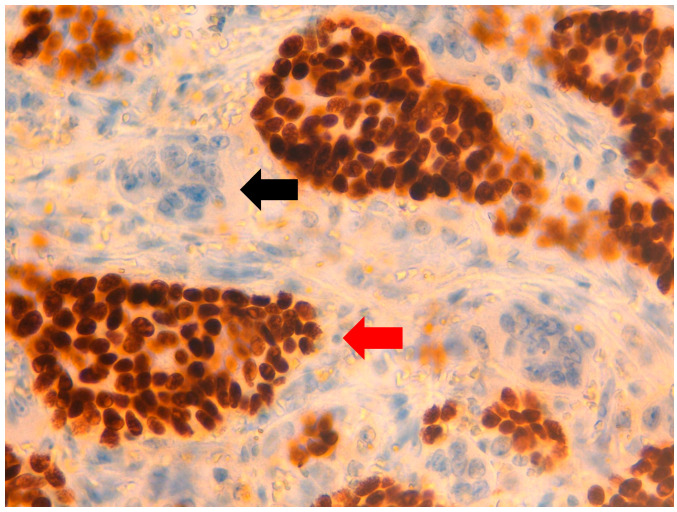
At 40× magnification. Immunohistochemical-positive reaction of neoplastic cells for progesterone receptor (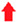), unlike OGCs which are progesterone receptor-negative (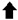).

**Figure 8:**
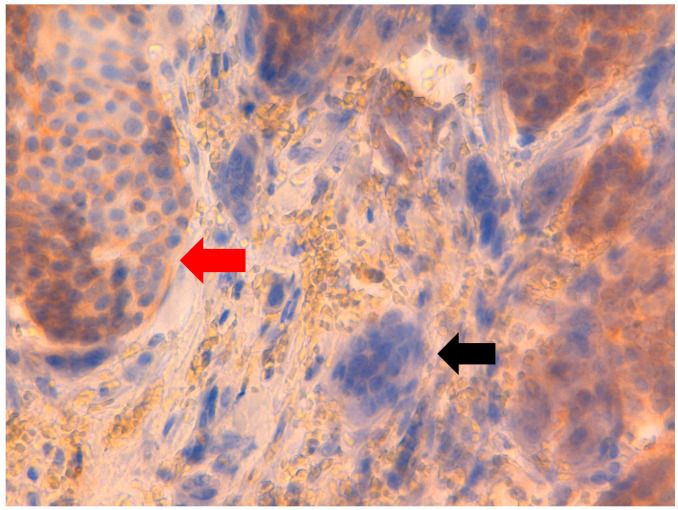
At 40× magnification. Immunohistochemical-negative reaction of neoplastic cells for human epidermal growth factor receptor 2 (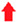) and OGCs (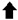).

**Table 1:** Immunohistochemical findings in invasive ductal carcinoma of the breast with osteoclast-like giant cells.

	Cancer Cells	Osteoclast-Like Giant Cells
***Estrogen Receptor***	+	-
***Progesterone Receptor***	+	-
***Human Epidermal Growth Factor Receptor 2***	-	-
***E-cadherin***	+	-
***CD68***	-	+
***CD163***	-	+

+: positive; -: negative.

## Data Availability

Data is contained within the article.
